# *IRX3* Overexpression Enhances *Ucp1* Expression *In Vivo*

**DOI:** 10.3389/fendo.2021.634191

**Published:** 2021-03-10

**Authors:** Zhiyin Zhang, Qihan Wu, Yang He, Peng Lu, Danjie Li, Minglan Yang, Weiqiong Gu, Ruixin Liu, Jie Hong, Jiqiu Wang

**Affiliations:** ^1^ Department of Endocrine and Metabolic Diseases, Shanghai Institute of Endocrine and Metabolic Diseases, Ruijin Hospital, Shanghai Jiao Tong University School of Medicine, Shanghai, China; ^2^ Shanghai National Clinical Research Center for Metabolic Diseases, Key Laboratory for Endocrine and Metabolic Diseases of the National Health Commission of the PR China, Shanghai National Center for Translational Medicine, Ruijin Hospital, Shanghai Jiao Tong University School of Medicine, Shanghai, China

**Keywords:** IRX3, Ucp1, thermogenesis, obesity, overexpression

## Abstract

**Objective:**

The Iroquois homeobox 3 (*IRX3*) gene was recently reported to be a functional downstream target of a common polymorphism in the *FTO* gene, which encodes an obesity-associated protein; however, the role of *IRX3* in energy expenditure remains unclear. Studies have revealed that the overexpression of a dominant–negative form of IRX3 in the mouse hypothalamus and adipose tissue promoted energy expenditure by enhancing brown/browning activities. Meanwhile, we and others recently demonstrated that *IRX3* knockdown impaired the browning program of primary preadipocytes *in vitro*. In this study, we aimed to further clarify the effects of overexpressing human *IRX3* (h*IRX3*) on brown/beige adipose tissues *in vivo*.

**Methods:**

Brown/beige adipocyte-specific h*IRX3*-overexpressing mice were generated and the browning program of white adipose tissues was induced by both chronic cold stimulation and CL316,243 injection. Body weight, fat mass, lean mass, and energy expenditure were measured, while morphological changes and the expression of thermogenesis-related genes in adipose tissue were analyzed. Moreover, the browning capacity of primary preadipocytes derived from h*IRX3*-overexpressing mice was assessed. RNA sequencing was also employed to investigate the effect of h*IRX3* on the expression of thermogenesis-related genes.

**Results:**

h*IRX3* overexpression in embryonic brown/beige adipose tissues (*Rosa26* ^h^ *^IRX3^*;*Ucp1-*Cre) led to increased energy expenditure, decreased fat mass, and a lean body phenotype. After acute cold exposure or CL316,243 stimulation, brown/beige tissue h*IRX3*-overexpressing mice showed an increase in *Ucp1* expression. Consistent with this, induced h*IRX3* overexpression in adult mice (*Rosa26* ^h^ *^IRX3^*;*Ucp1-*Cre^ERT2^) also promoted a moderate increase in *Ucp1* expression. *Ex vitro* experiments further revealed that h*IRX3* overexpression induced by *Ucp1*-driven Cre recombinase activity upregulated brown/beige adipocytes *Ucp1* expression and oxygen consumption rate (OCR). RNA sequencing analyses indicated that h*IRX3* overexpression in brown adipocytes enhanced brown fat cell differentiation, glycolysis, and gluconeogenesis.

**Conclusion:**

Consistent with the *in vitro* findings, brown/beige adipocyte-specific overexpression of h*IRX3* promoted *Ucp1* expression and thermogenesis, while reducing fat mass.

## Introduction

*Iroquois homeobox gene 3* (*IRX3*) encodes a transcription factor of the Iroquois family of homeodomain-containing proteins (1). *IRX3* is initially expressed during embryogenesis, and is involved in the development and patterning of multiple tissues, including the nervous system, heart, and skeleton (2). Recent studies have indicated that *IRX3*, together with its homolog *IRX5*, may also have a role in energy balance and adiposity by regulating thermogenesis in brown adipose tissue (BAT) and the browning program in white adipose tissue (WAT). WAT stores energy in the form of triglycerides under excess caloric intake, whereas BAT dissipates energy through the activity of the inner mitochondrial membrane-localized uncoupled protein 1 (UCP1) to maintain body temperature hemostasis (3–5). Evidence for several genome-wide association studies (GWAS) has indicated that several common single nucleotide polymorphism (SNP) variants, such as rs1421085 and rs9930506, located in the first intron of fat mass and obesity associated (*FTO*) gene, are strongly associated with an increased risk of obesity (6–8), and the functional loci and biological targets associated with these variants are largely unknown.

Recently, *IRX3* and *IRX5*, but not *FTO*, were proposed to function as targets of the rs1421085 variant in thermogenesis (9). However, whether IRX3 acts as an activator or repressor of thermogenesis, where the functional target(s) are located, and whether other candidate genes including *FTO*, *IRX5*, and *Rpgrip1l* were involved in thermogenesis regulation of rs1421085 (10, 11), remain unclear. Another study reported that the hypothalamic overexpression of a dominant–negative form of IRX3 (EnR*-Irx3*) (12), which can increase instead of suppress the transcriptional activities of the IRX3 protein in certain contexts (13), induced a lean body phenotype accompanied by an enhanced WAT browning capacity and activation of BAT (12). Additionally, the overexpression of this “dominant–negative” form of IRX3 (EnR*-Irx3;aP2-*Cre) in adipose tissue also induced a lean body phenotype with marked browning changes in WATs (14). In contrast, a recent study demonstrated that partial (approximately 50%) inhibition of endogenous hypothalamic IRX3 expression reduced thermogenesis in peripheral BAT and increased diet-induced body mass gain, thereby exacerbating obesity (15). We have previously also provided evidence that *Irx3* knockdown can impair the thermogenic capacities of induced brown and beige adipocytes derived from preadipocytes of mouse inguinal WAT (iWAT) and BAT, and human subcutaneous WAT, respectively; and that missense mutations in *IRX3* identified in humans markedly reduced the transcription of *UCP1 in vitro* (16). Consistent with these observations, another group recently showed that *Irx3* knockout in mouse preadipocytes impaired both the early and late stages of adipogenic differentiation to beige adipocytes and preadipocyte mitochondrial respiration (17). These contradictory data highlight the importance of identifying the precise roles of human *IRX3* (wild type) in brown/beige adipocytes *in vivo*.

To this end, we generated two brown/beige adipocyte-specific h*IRX3*-overexpressing mouse models by crossing *Rosa26* ^h^ *^IRX3^* knock-in mice with *Ucp1-*Cre mice, resulting in the continuous induced expression of h*IRX3* from the embryonic stage, as well as with *Ucp1-*Cre^ERT2^ mice, which expressed h*IRX3* from adulthood following tamoxifen (TMX) injection. In *Ucp1-*Cre mice, h*IRX3* overexpression in BAT led to increased energy expenditure, decreased fat mass, and a lean body phenotype, while h*IRX3* overexpression in adulthood induced a subtle increase in thermogenesis after stimulation with a β3-AR agonist. Furthermore, h*IRX3* overexpression significantly enhanced beige adipocyte differentiation concomitant with increased *Ucp1* expression. Together, our results revealed that h*IRX3* overexpression can promote thermogenesis in brown/beige adipose tissue *in vivo*, and provide a more comprehensive understanding of the role of IRX3 in energy balance and obesity.

## Materials and Methods

### Animal Models

To generate *Rosa26* ^h^ *^IRX3^* (h*IRX3* knock-in) mice, a human *IRX3* cDNA-polyA cassette (GenBank accession number: NM_024336.2; Ensembl: ENSG00000177508) was cloned into intron 1 of the *Rosa26* locus, and CAG-loxP-stop-loxP was inserted upstream of the cassette. *Ucp1-*Cre mice were obtained from the Jackson Laboratory (Jax no. 024670). To generate *Ucp1-*Cre^ERT2^ mice, a CreERT2-IRES-EGFP-PA cassette was knocked-in downstream of the ATG start codon of the m*Ucp1* gene such that the expression of CreERT2 and EGFP were under the control of m*Ucp1* regulatory sequences. Mouse genomic fragments were amplified with high-fidelity Taq DNA polymerase and were assembled into a targeting vector, together with recombination sites and selection markers, as indicated in the vector map in **Supplementary Figure 1A**. The final sequence of the targeting vector is shown in **Supplementary Figure 1B**. The constitutive h*IRX3* knock-in allele was obtained after Flp-mediated recombination (**Supplementary Figure 1C**). C57BL/6 embryonic stem cells were used for gene targeting. Genotypes were verified by PCR (**Supplementary Figure 1D**). Maximal Cre recombinase mRNA expression was seen in BAT (**Supplementary Figures 1E, F**). *Ucp1-*Cre;*Rosa26* ^h^ *^IRX3^* (U-IRX3^ov^) and *Ucp1-*Cre^ERT2^;*Rosa26* ^h^ *^IRX3^* (iU-IRX3^ov^) mice were generated using the Cre/loxP system. *Rosa26* ^h^ *^IRX3^* was used as a control with U-IRX3^ov^ mice. As *Ucp1-*Cre^ERT2^ showed an approximately 50% decrease in Ucp1 protein expression compared with that of endogenous *Ucp1* (**Supplementary Figure 1G**), we used *Rosa26* ^wild type(WT)^;*Ucp1-*Cre^ERT2^ as the control for iU-IRX3^ov^. iU-IRX3^ov^ mice and control littermates were treated intraperitoneally with TMX at a dose of 100 mg/(kg·day^−1^) to induce h*IRX3* expression (**Supplementary Figure 1H-K**). All animal procedures were approved by the Animal Care Committee of Shanghai Jiao Tong University School of Medicine and followed the guide for the care and use of laboratory animals.

### Cold Exposure and CL316,243 Injection

For cold exposure, mice were placed individually in a room with the temperature set at 4°C for 7 days. The animals had free access to food and water during this period. Their core body temperature was measured using a rectal probe (Physitemp Instruments Inc., USA). CL316,243 (Sigma-Aldrich, USA) was injected interperitoneally at a dose of 1 mg/(kg·day^−1^), and the injection protocol was described in the results part for details.

### Measurement of Fat/Lean Mass and Whole-Body Energy Metabolism

The fat mass and lean mass of each mouse was measured using an EchoMRI-100H (EchoMRI, USA). The mice were placed in a Comprehensive Laboratory Animal Monitoring System (CLAMS, Columbus Instruments, USA) for the evaluation of whole-body energy metabolism. Oxygen and carbon dioxide consumption, as well as activity, was continuously measured for two days. The respiratory exchange ratio (RER) and energy expenditure were calculated based on the oxygen and carbon dioxide data and were normalized to body weight (18).

### Morphological Analysis

BATs and WATs were isolated, fixed in 4% paraformaldehyde, embedded in paraffin, and sliced into 5-μm (iWAT, eWAT) or 3-μm (BAT) sections for hematoxylin and eosin (H&E) staining. Images were captured under a microscope (Olympus, Japan). Pictures were scanned by Digital Pathology Slide Scanner (KF-PRO-120).

### Isolation of the Stromal Vascular Fraction and Brown/Beige Adipocyte Differentiation *In Vitro*

The stromal vascular fractions (SVFs) were isolated from the BAT and iWAT of five-week-old U-IRX3^ov^ and control mice and then induced to fully differentiate into brown/beige adipocytes as previously described (5). In brief, the fat pads were isolated, cut into pieces, and digested with type II collagenase (Sigma) at 37°C for 30 min followed by quenching with DMEM/F12 supplemented with 10% FBS. The suspended samples were filtered using a 40-μm strainer (BD, USA) and then plated on culture dishes. The SVFs were first grown to 100% confluence in DMEM/F12 supplemented with 10% FBS (plus 1% penicillin/streptomycin and 1 mM L-glutamine), and then these primary preadipocytes were differentiated into brown/beige adipocytes in a cocktail containing 5 μg/ml insulin (Eli Lilly, USA), 1 μM dexamethasone, 1 μM rosiglitazone, 1 μM triiodothyronine (T_3_), and 0.5 mM IBMX (all Sigma–Aldrich, USA) for two days, and subsequently in medium with insulin, rosiglitazone, and T_3_ for another six days.

### Oil Red O Staining

After eight days of induction, mature adipocytes were stained with Oil Red O. In brief, the cells were fixed in 4% paraformaldehyde for 30 min, rinsed, air-dried, and incubated with Oil Red O (Nanjing Jiancheng Bioengineering Institute, China) for 30 min. Images were captured under a microscope (Olympus).

### Measurement of the Oxygen Consumption Rate

SVFs were seeded in an XF24 V28 microplate (Agilent Technologies, USA) coated with poly-L-lysine. The induction protocol was as described in section 2.5. The oxygen consumption rate (OCR) was measured at induction day 4 using an XF24 analyzer (Agilent Technologies) following the manufacturer’s instructions. Briefly, the induced brown/beige adipocytes were washed with Seahorse assay medium, consisting of XF DMEM supplemented with 10 mM XF glucose, 1 mM XF pyruvate, and 2 mM XF L-glutamine, followed by incubation with 525 μl of assay medium at 37°C in an incubator without CO_2_ (Agilent Technologies) for 1 h. Respiratory inhibitors (75 μl) were loaded into the injection port to final concentrations of 1 mg/ml oligomycin, 2 mM FCCP, 0.5 mM antimycin A, and 0.5 mg/ml rotenone to detect uncoupled respiration, maximal respiration, and nonmitochondrial respiration, respectively. The final OCR results were standardized to the total protein content. The results are representative of at least three independent experiments.

### RNA Extraction and Real-Time PCR Analysis

Total RNA was extracted from cultured cells or frozen adipose tissue using the Eastep Super Total RNA Extraction Kit (Promega (Beijing) Biotech Co., China). The absorbance ratio at 260/280 nm and the RNA concentration of each sample were detected using a NanoDrop ND2000 spectrophotometer (Thermo Scientific). Reverse transcription was performed using the PrimeScript Reverse Transcript Master Mix (TaKaRa, Japan). qPCR was performed using a QuantStudio Dx Real-Time PCR Instrument (Applied Biosystems). The comparative ΔΔCt method was used to evaluate the relative mRNA levels; 36B4 served as the reference gene (**Supplementary Table 1**).

### RNA Sequencing and Analysis

RNA sequencing was performed by NovelBio, Shanghai, China. The RNA quality was assessed using an Agilent 2200 and the RNA was stored at −80°C. RNA with an RNA integrity number (RIN) >7 was considered acceptable for cDNA library construction. cDNA libraries were constructed for each RNA sample using the TruSeq Stranded mRNA Library Prep Kit (Illumina) according to the manufacturer’s instructions. The libraries were quality controlled with Agilent 2200 and sequenced by HiSeq X (Illumina) as 150-bp paired-end reads. For the analysis of differentially expressed genes, *P*-value and false discovery rate (FDR) analysis were subjected to the following criteria: i) Fold change (FC) >2 or <0.5; ii) *P*-value <0.05, FDR <0.05. Fisher’s exact test was applied to identify significant GO categories and KEGG pathways (*P*-value <0.05). The approach for gene set enrichment analysis (GSEA) was in accordance with that previously reported (19). Genes were considered to be significantly differentially expressed when the FDR was less than 0.05 and the log_2_FC was more than 1.

### Protein Preparation and Western Blot Analysis

Total protein was isolated using RIPA lysis buffer (Biocolors, China) with a protease inhibitor cocktail (Sigma). Western blotting was performed as previously described (5). The following antibodies were used: anti-IRX3 (ab174307, Abcam), anti-UCP1 (ab10983, Abcam), anti-Hsp90 (Cell Signaling Technology, 4877s), and anti-PGC-1α (Abcam, ab54481). The results are representative of at least three independent experiments.

### Statistical Analysis

Data are shown as means ± S.E.M, and the results were compared by two-tailed *t*-tests. A *P*-value <0.05 was considered to be significantly different. Spearman’s correlation analysis was performed to examine the associations between the expression of h*IRX3* and m*Ucp1*. For molecular experiments, data were generated from three independent experiments. Analyses were undertaken with GraphPad Prism version 8.2.1 (279) (GraphPad Software, Inc., La Jolla, CA, USA).

## Results

### Brown/Beige Adipocyte-Specific Overexpression of h*IRX3* Increased Energy Expenditure and Induced a Lean Body Phenotype *In Vivo*

**Figure 1:**
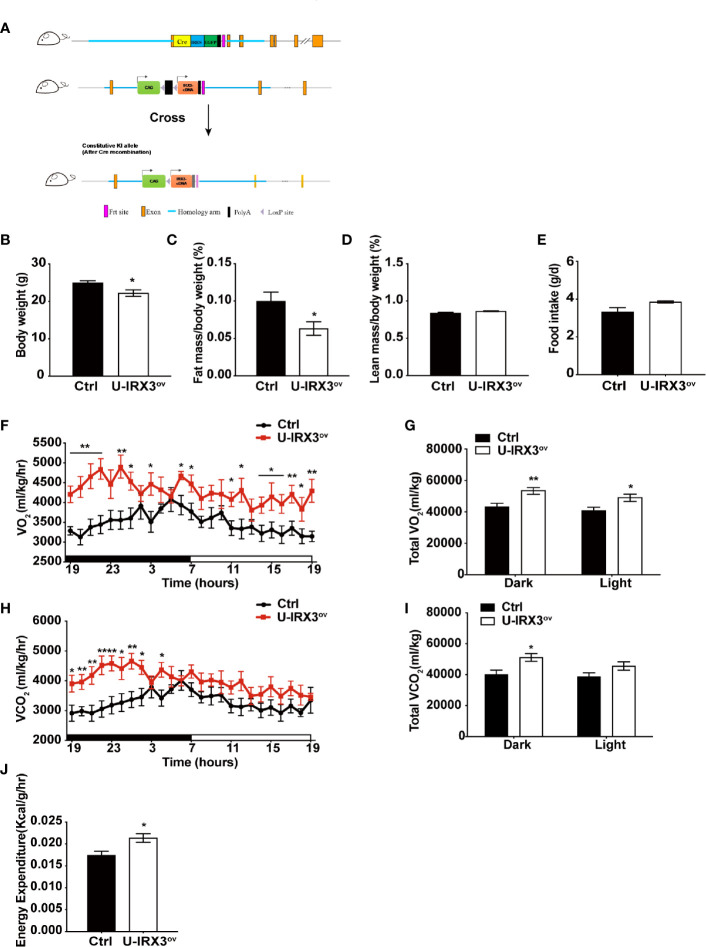
Brown/beige adipocyte-specific h*IRX3* overexpression increases energy expenditure and induces a lean body phenotype in male mice. **(A)** Schematic diagram of the generation of U-IRX3^ov^ (*Rosa26*-loxP-stop-loxP-h*IRX3*;*Ucp1*-Cre) mice. **(B**–**D)** Body weight **(B)**, fat mass percentage **(C)**, and lean mass percentage **(D)** of male U-IRX3^ov^ and control mice at 10 weeks of age (*n* = 6~8). **(E)** Average daily food intake of male U-IRX3^ov^ and control mice at 10 weeks of age (average of 3 individual measurements). **(F**–**J)** Whole-body oxygen (O_2_) consumption per hour **(F)** and per 12 h **(G)**, carbon dioxide (CO_2_) production per hour **(H)** and per 12 h **(I)**, and energy expenditure **(J)** of 10-week-old U-IRX3^ov^ and control mice during a 24-h period (*n* = 6~8). Statistics were standardized by body weight. Data are shown as means ± SEM. *P < 0.05, **P < 0.01.

To clarify the physiological and biological roles of h*IRX3* in thermogenesis in brown/browning adipose tissues, we generated a *Ucp1-*Cre-driven h*IRX3* overexpression mouse model (*Ucp1-*Cre; *Rosa26* ^h^ *^IRX3^*, referred to as U-IRX3^ov^) (**Figure 1A**). When fed a normal chow diet, both male and female U-IRX3^ov^ mice gained substantially less body weight than the controls (*Rosa26* ^h^ *^IRX3^*) at 10 weeks of age (**Figure 1B** and **Supplementary Figures 2A–C**). Body composition analysis revealed a lower fat mass percentage in U-IRX3^ov^ mice when compared with controls; however, there was no significant difference in lean mass percentage between the two genotypes (**Figures 1C, D** and **Supplementary Figures 2D, E**). To test for potential alterations in energy balance, we undertook a comprehensive evaluation of the food intake, physical activities, and energy expenditure of the mice. We found that, with comparable daily food intake (**Figure 1E** and **Supplementary Figure 2F**), U-IRX3^ov^ mice had greater O_2_ consumption, increased CO_2_ production, and greater total energy expenditure, especially at night, compared with controls (**Figures 1F–I**). U-IRX3^ov^ mice showed a slight increase in physical activities in few hours of a day (*X-*and *Y*-axis), although the total increase was not statistically significant (**Supplementary Figures 2G–I**). Importantly, U-IRX3^ov^ mice displayed higher average energy expenditure per hour compared with controls (**Figure 1J**). These findings suggested that U-IRX3^ov^ mice gained less fat mass, which was likely due to increased energy expenditure.

### Overexpression of h*IRX3* Enhances Thermogenesis-Associated Gene Expression Following Chronic Cold or CL316,243 Stimulation

**Figure 2:**
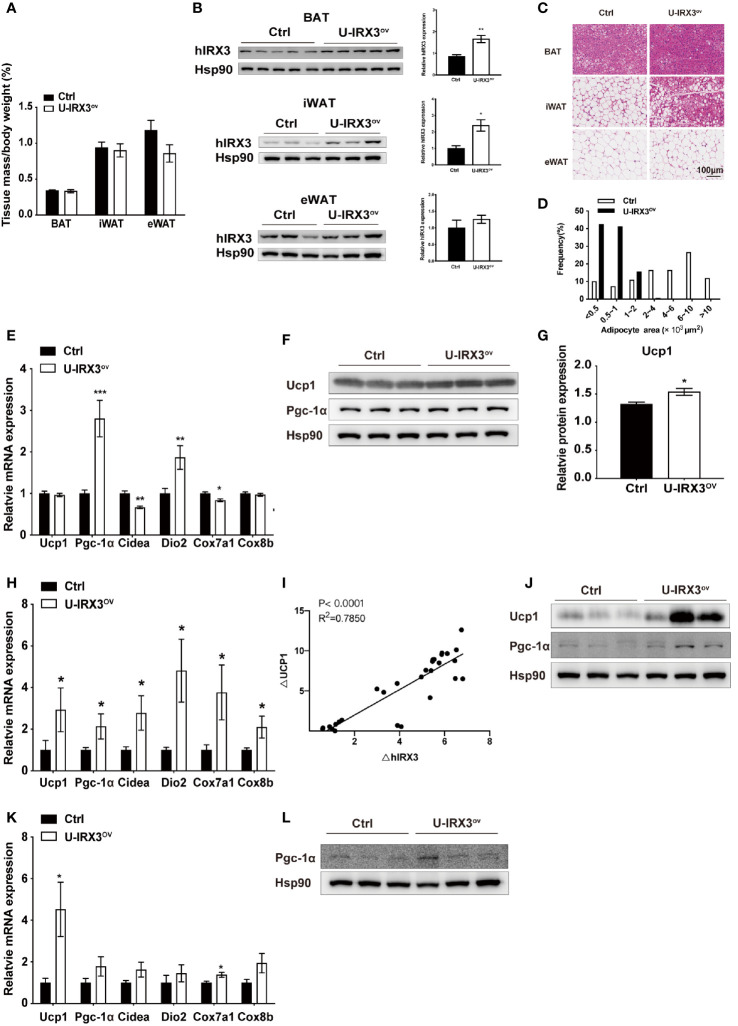
The overexpression of h*IRX3* from the embryonic stage enhances cold-induced thermogenesis. **(A**–**L)** Ten-week-old U-IRX3^ov^ and control mice were placed in a cold room at 4°C for 7 days. Tissue mass percentage **(A)** and protein levels of overexpressed hIRX3 in BAT, iWAT, and eWAT of the two groups of mice (*n* = 3~5). **(B)** Representative images of hematoxylin and eosin (H&E) staining of BAT (top), iWAT (middle), and eWAT (bottom) for the two groups of mice **(C)**. **(D)** Adipocyte size distribution of the eWAT in **(C)**. Scale bars, 100 μm. **(E)** The mRNA expression levels of thermogenesis-related genes in the BAT of the two groups of mice (*n* = 6~8). **(F)** The protein levels of UCP1 and PGC-1α in the BAT of the two groups of mice (*n* = 3). **(G)** The UCP1 protein expression level relative to that of Hsp90 in **(F)**. **(H)** The mRNA expression levels of thermogenesis-related genes in the iWAT of the two groups of mice (*n* = 6~8). **(I)** Linear regression analyses of hIRX3 and Ucp1 RNA expression level of male (n=13) and female(n=16) U-IRX3^ov^ and control mice **(J)** The protein levels of UCP1 and PGC-1α in the iWAT of the two groups of mice (*n* = 3). **(K)** The mRNA expression levels of thermogenesis-related genes in the eWAT of the two groups of mice (*n* = 6~8). **(L)** The protein levels PGC-1α in the eWAT of the two groups of mice (*n* = 3). Data are shown as means ± SEM. *P < 0.05.

Next, to test the response of the mice to acute and chronic cold, we subjected the two groups of mice to cold treatment at 4°C. No significant differences in body temperature were observed during the first 6 h (**Supplementary Figure 2J**). However, after seven days of cold stimulation, the mice showed a subtle, but statistically insignificant, decrease in eWAT mass percentage (*P* = 0.08) (**Figure 2A**). With moderate h*IRX3* overexpression in inguinal WAT (iWAT) and BAT (**Figure 2B** and **Supplementary Figure 2K**), U-IRX3^ov^ mice showed marked morphological changes in iWAT, characterized by a more condensed texture with markedly increased number of smaller lipid droplets, as well as a significantly reduced droplet content in BAT, and an increase in the percentage of smaller adipocytes in eWAT compared with those in control mice (**Figures 2C, D**). We next examined the expression levels of thermogenesis-related genes in three adipose tissues, and found that the mRNA levels of *Pgc-1α* and *Dio2* were increased in the BAT of U-IRX3^ov^ mice (**Figure 2E**). Although the mRNA levels of *Ucp1* were unchanged, the BAT of U-IRX3^ov^ mice exhibited a small but substantial increase in UCP1 protein levels (**Figures 2F, G**). For iWAT, a marked increase in the mRNA expression levels of *Ucp1*, *Cidea*, *Dio2*, *Cox7a1*, and *Cox8b* was observed in U-IRX3^ov^ mice (**Figure 2H**). Consistent with our previous *in vitro* findings, h*IRX3* mRNA expression *in vivo* was also positively correlated with that of *Ucp1* (**Figure 2I**). The protein levels of UCP1 and PGC-1α in iWAT were also increased in the iWAT of U-IRX3^ov^ mice (**Figure 2J**). However, only the mRNA levels of *Ucp1* and *Cox8b*, and the protein levels of PGC-1α, showed increased expression in the eWAT of U-IRX3^ov^ mice (**Figures 2K, L**).

To further validate the promotive effect of h*IRX3* on thermogenesis in BAT, we intraperitoneally injected CL316,243, an agonist of the β3-adrenergic receptor (β3-AR), into female U-IRX3^ov^ and littermate control mice to activate BAT and induce the browning process. Male mice showed no significant change in mass under cold treatment; however, female U-IRX3^ov^ mice displayed a reduction in BAT, iWAT, and gWAT (gonadal WAT) content (**Supplementary Figure 3A**). Histomorphological analysis revealed that the adipocytes were smaller and more condensed in all these adipose tissues (**Supplementary Figures 3B–D**). Both the mRNA and protein expression levels of *Ucp1* and *Pgc-1α* were increased in the BAT of female U-IRX3^ov^ mice (**Supplementary Figures 3E, F**). The expression of thermogenesis-related genes, such as *Ucp1*, *Pgc-1α*, *Cidea*, and *Dio2*, was enhanced in the iWAT of U-IRX3^ov^ mice (**Supplementary Figure 3G**). Similarly, UCP1 protein levels showed an increasing trend in the iWAT of U-IRX3^ov^ mice (**Supplementary Figure 3H**). Collectively, these results indicated that the overexpression of h*IRX3* increased both cold treatment- and CL316,243-induced thermogenesis in brown/beige adipose tissues *in vivo*.

### Overexpression of hIRX3 in Adulthood Enhanced β3-AR Agonist-Induced Thermogenesis

**Figure 3:**
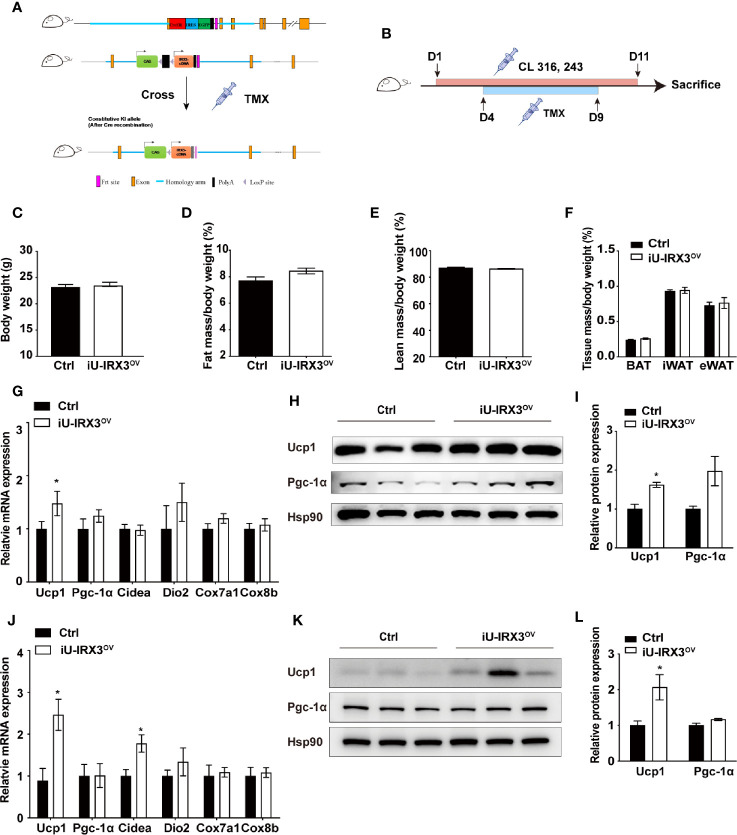
Overexpressing hIRX3 after adulthood increased the expression of thermogenesis-related genes under β3-AR agonist (CL316,243) stimulation. **(A)** Schematic representation of the strategy to generate iU-IRX3^ov^ mice. **(B)** Timeline of CL316,243 and tamoxifen (TMX) injection in eight-week-old male iU-IRX3^ov^ and control mice (*n* = 9~10). **(C**–**F)** Body weight **(C)**, fat mass percentage **(D)**, lean mass percentage **(E)**, and tissue mass percentage of BAT, iWAT, and eWAT **(F)** in male iU-IRX3^ov^ and control mice. **(G)** The relative mRNA expression levels of *Ucp1* and other thermogenesis-related genes in the iWAT of iU-IRX3^ov^ mice(*n* = 9~10). **(H, I)** Images and quantitative values of UCP1 and PGC-1α protein levels in iWAT (*n* = 3). **(J)** The relative mRNA expression levels of *Ucp1* and other thermogenesis-related genes in eWAT (*n* = 9~10). **(K, L)** Images and quantitative values of UCP1 and PGC-1α protein levels in eWAT (*n* = 3). Data are shown as means ± SEM. *P < 0.05.

The protein expression of UCP1 in BAT first appears in late gestation and then rapidly increases at birth (20), allowing *Ucp1* promoter-driven Cre recombinase to excise loxP-flanked (floxed) sequences (STOP in this study) and theoretically induce h*IRX3* overexpression prepartum. A recent study demonstrated that *Irx3* ablation in mouse preadipocytes attenuated the proliferation and early differentiation of beige adipocytes *in vitro* (17). To avoid the nonspecific consequences of overexpressing h*IRX3* in brown preadipocytes, and to investigate the effects of transient h*IRX3* overexpression in mature beige adipocytes in adult mice, we generated a TMX-inducible h*IRX3* overexpressing mouse line (*Ucp1-*Cre^ERT2^; *Rosa26* ^h^ *^IRX3^*, iU-IRX3^ov^) by crossing *Rosa26* ^h^ *^IRX3^* mice with *Ucp1-*Cre^ERT2^ mice (**Figure 3A**). Then, we treated eight-week-old male iU-IRX3^ov^ and control mice with TMX (i.p. once/day) for 5 days during the 10-days CL316,243 injection interval (**Figure 3B**). The overexpression of h*IRX3* in adipose tissue was validated by qPCR and Western-blot (**Supplementary Figures 1H–K**). No differences in body weight, fat mass percentage, lean mass percentage, or adipose tissue weight were observed between the two groups (**Figures 3C–F**). In the iWAT of iU-IRX3^ov^ mice, there was a small but significant increase in *Ucp1* mRNA and protein levels (p<0.05), while the expression of other thermogenesis-related genes showed an increasing, but insignificant, trend (**Figures 3G–I**). Interestingly, the mRNA expression of *Ucp1*, *Pgc-1α*, and *Cidea* was significantly increased in the eWAT of iU-IRX3^ov^ mice when compared with controls (**Figure 3J**), which was accompanied by an increase in UCP1 protein levels (**Figures 3K, L**). Combined, these results indicated that the temporary overexpression of h*IRX3* in WAT of adult mice, and especially in eWAT, can enhance thermogenesis, but with a relatively subtle effect when compared with that seen in U-IRX3^ov^ mice.

### Overexpression of h*IRX3* Increases Thermogenesis in Brown/Beige Adipocytes *In Vitro*

**Figure 4:**
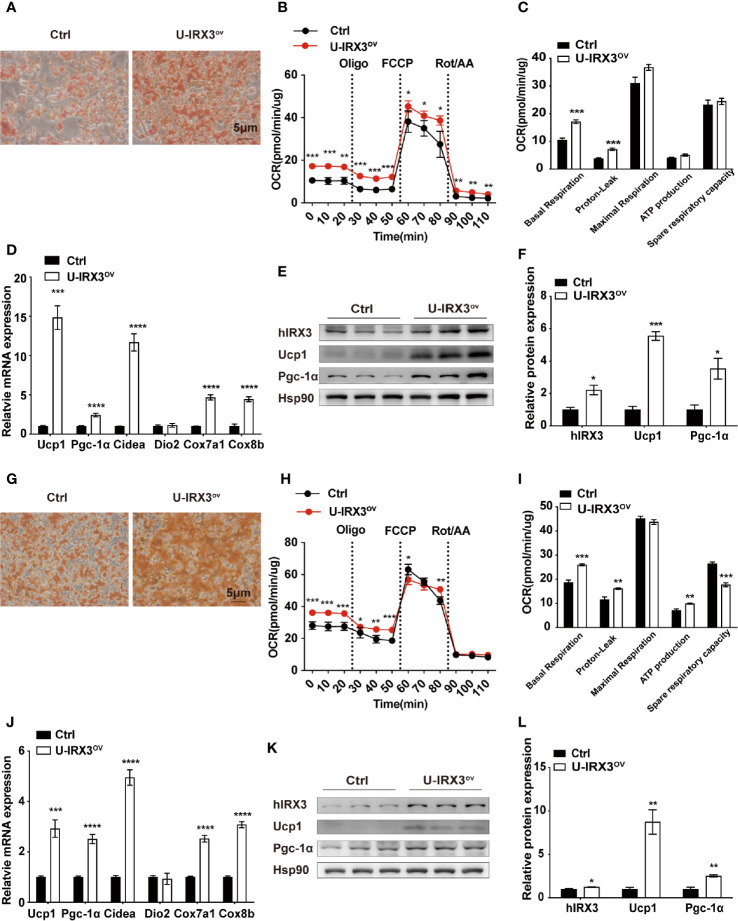
The overexpression of h*IRX3* increases thermogenesis in brown/beige adipocytes *in vitro*. **(A–F)** The overexpression of h*IRX3* in stromal vascular fractions (SVFs) derived from BAT of U-IRX3^ov^ and control mice. Oil Red O staining **(A)**, oxygen consumption rate (OCR) **(B, C)**, mRNA expression levels of thermogenesis-related genes **(D)**, and the protein expression **(E)** and their quantitative values **(F)**, including hIRX3, PGC-1a, and UCP1, in induced brown adipocytes after five days of differentiation. **(G–L)** The overexpression of h*IRX3* in SVFs derived from the iWAT of U-IRX3^ov^ and control mice. Oil Red O staining of beige adipocytes after eight days of differentiation **(G)**; the OCR of beige adipocytes after eight days of differentiation **(H, I)**; the mRNA expression levels of thermogenesis-related genes **(J)**, and the protein expression **(K)** and their quantitative values **(L)**, including hIRX3, PGC-1a, and UCP1, in induced beige adipocytes after eight days of differentiation. Data are shown as means ± SEM. *P < 0.05, **P < 0.01, ***P < 0.001, ****P < 0.0001.

We next investigated the effects of h*IRX3* overexpression on the differentiation and thermogenic capacities of induced brown and beige adipocytes obtained from primary preadipocyte SVFs. Over expression of hIRX3 was proved by qPCR and Western-blot **(** **Figures 4E, K** and **Supplementary Figure 3I**). BAT SVF derived from U-IRX3^ov^ mice showed markedly enhanced adipogenic differentiation capacity compared with controls, as evidenced by the increased number of multilocular lipid droplets in Oil Red O staining (**Figure 4A**). We also assessed the thermogenic capacity of induced brown adipocytes, represented by the mitochondrial OCR. Compared with controls, there was a significant increase in basal respiration and proton leak, as well as an increasing trend for the maximal respiration and ATP production capacity of the induced brown adipocytes derived from U-IRX3^ov^ mice. This suggested that h*IRX3* overexpression led to an increase in thermogenesis (**Figures 4B, C**). Meanwhile, the mRNA levels of thermogenesis-related genes, such as *Ucp1*, *Pgc-1α*, *Cidea*, *Cox7a1*, and *Cox8b*, were significantly increased in induced brown adipocytes derived from U-IRX3^ov^ mice (**Figure 4D**). Consistent with these results, the protein levels of UCP1 and PGC-1α were also enhanced by h*IRX3* overexpression (**Figures 4E, F**). Additionally, when the SVFs obtained from the iWAT of U-IRX3^ov^ and control mice were induced to beige adipocytes *in vitro*, we found that adipogenesis, thermogenic capacity, and expression of thermogenesis-related genes were all increased in the U-IRX3^ov^ group (**Figures 4G–L**). Together, these results indicated that the thermogenic capacity can be improved in brown and beige adipocytes following h*IRX3* overexpression mediated by *Ucp1* promoter-driven Cre recombinase activity *in vitro*.

### h*IRX3* Enhances Thermogenesis Through Increasing *Ucp1* Expression

**Figure 5:**
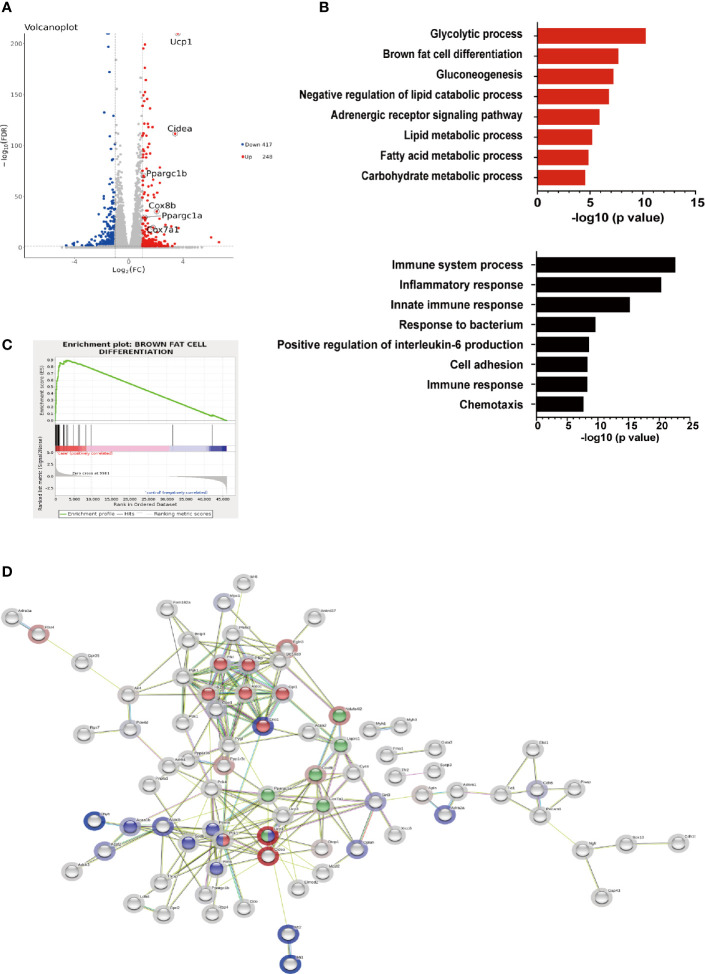
The gene expression profile of h*IRX3*-overexpressing brown adipocytes. **(A–D)** After eight days of differentiation, RNA-seq analysis was performed on induced brown adipocytes derived from BAT of U-IRX3^ov^ and littermate control mice (*n* = 3). **(A)** Volcano plot of the differentially expressed genes; red: upregulated, blue: downregulated. The *x*-axis represents the log-fold change, and the *y*-axis represents the −log10 of the false discovery rate (FDR). Several key thermogenesis-related markers are circled. **(B)** The top gene ontology (GO) biological process terms enriched (*P* < 0.05, Fisher’s test) among genes that show significantly higher (top 8, red) or lower (top 8, black) expression (*P* < 0.05, DESeq) in the U-IRX3^ov^ group relative to controls. **(C)** Gene set enrichment analysis (GSEA) of overlap between genes upregulated following h*IRX3* overexpression and the brown fat cell differentiation gene signature in the GO analysis. NES, normalized enrichment score; *p*, empirical *p*-value. **(D)** A protein–protein interaction network of the upregulated genes (*P* < 0.05, log2FC ≥1, group average count ≥50, *n* = 129) was constructed using the STRING database (https://string-db.org) applying an interaction score >0.4. The halo color represents the log2FC value from low (blue) to high (red). Gene clusters for the main KEGG pathways are shown based on node color (red for glycolysis/gluconeogenesis, blue for PPAR signaling pathway, green for thermogenesis).

To further elucidate the effects of hIRX3 overexpression on thermogenesis, we performed RNA-seq analysis on induced brown adipocytes derived from the SVFs of U-IRX3^ov^ and control mice. In total, we identified 665 differentially expressed genes (FC <2, FDR <0.05) between the U-IRX3^ov^ and control groups, 248 of which were upregulated and 417 downregulated (**Supplementary Tables 2** and **3**). Notably, and consistent with the qPCR findings, several genes annotated as being positively related to the browning program, such as *Ucp1*, *Cidea*, *Pgc-1α*, *Cox7a1*, and *Cox8b*, were markedly upregulated in the U-IRX3^ov^ group (**Figure 5A**). Gene Ontology (GO) analysis revealed that the upregulated genes were enriched in biological processes involved in brown fat cell differentiation, glycolysis, gluconeogenesis, and other metabolic process associated with energy expenditure; meanwhile, the downregulated genes were mainly associated with immune system process, inflammatory response, and cell adhesion (**Figure 5B**), processes that are usually suppressed during brown adipogenesis or thermogenesis (21–23). Gene set enrichment analysis (GSEA) further indicated a marked overlap between enriched genes and the gene signature activated during brown adipogenesis (**Figure 5C**). We then explored protein–protein connection of upregulated genes, and clustered connected genes which classified into the same KEGG pathway. The results showed the upregulated genes were enriched in pathways related to thermogenesis, oxidative phosphorylation, glycolysis/gluconeogenesis, and PPAR signaling (**Figure 5D**). Collectively, these data suggested that h*IRX3* overexpression in induced brown adipocytes can promote *Ucp1* expression, brown cell adipocyte differentiation, and thermogenesis.

## Discussion

In this study, we generated two genetically modified mouse models to clarify the effects of h*IRX3* overexpression *in vivo*, and present evidence that h*IRX3* overexpression in mouse brown/beige adipocytes leads to an enhancement of thermogenesis. Using the U-IRX3^ov^ line, in which h*IRX3* is continuously expressed from an early stage of life, we found that the functional abilities of BAT and the browning program of WAT were both enhanced, which resulted in the increased expression of thermogenesis-related genes (including *Ucp1*), increased energy expenditure, smaller lipid droplets, and lower fat mass percentage. Using the iU-IRX3^ov^ mouse line, in which the expression of h*IRX3* is transiently induced in adulthood, we identified a weak but similar increase in the expression of thermogenesis-related genes in iWAT and eWAT.

Several GWAS studies have demonstrated that genetic variations (SNPs) within the first and second introns of the *FTO* gene are positively associated with an increased risk of obesity (6–8, 24–26). Among these SNPs, one variant—rs1421085—was recently identified as an underlying cause of adiposity through increasing *IRX3* expression *via* long-range chromatin interaction (9, 12). However, whether *IRX3* augments or attenuates the thermogenic capacities of brown/beige adipocytes remains unclear (27–29). In contrast to the findings that IRX3 is an inhibitor of the browning progress, we previously reported that *IRX3* expression is elevated in human and mouse brown/beige adipocytes, and that the browning program of adipocytes is repressed when *IRX3* is knockdown *in vitro* (16, 17). Importantly, we found that *IRX3* could upregulate the transcription of *Ucp1* by specific binding to the ACATGTGT motif (−3470 to −3463 bp) upstream of the transcription starting site of the mouse *Ucp1* gene. In this study, we aimed to further clarify the role of the human IRX3 in thermogenesis and its effect on *Ucp1* expression, especially in *Ucp1*-expressing brown/beige adipocytes. We found that h*IRX3* enhanced thermogenesis in both h*IRX3*-overexpression mouse models (noninducible and TMX-inducible). Of note, thermogenesis was more prominent in U-IRX3^ov^ (noninducible *Ucp1-*Cre) mice than in iU-IRX3^ov^ (inducible *Ucp1-*Cre^ERT2^) mice. This difference could be attributed to the temporal, spatial, and dosage differences in h*IRX3* expression between the two models. First, h*IRX3* was expected to be expressed in brown adipocytes of U-IRX3^ov^ mice shortly after birth, similar to that observed for normal *Ucp1* expression (30), whereas h*IRX3* expression was only relatively weakly induced in brown adipocytes of adult iU-IRX3^ov^ mice after TMX injection. Second, there may be heterogeneity in the spatial and quantitative expression of Cre recombinase: the ectogenic *Ucp1*-Cre construct was inserted spontaneously, thus Cre expression would have been extensively induced (might not restricted to brown and beige adipose tissue), whereas the endogenous expression of *Ucp1* would not be affected (31). On the other hand, in *Ucp1-*Cre^ERT2^, the Cre sequence was directly inserted into exon 1 of *Ucp1*, which means the Cre only expressed where endogenous Ucp1 appeared, but at the same time impaired endogenous *Ucp1* expression and led to *Ucp1* protein reduced by half. Thus the Cre recombinase in the *Ucp1-*Cre^ERT2^ model likely showed weak gene-editing activity. Notably, the effect of *IRX3* on thermogenesis may change according to the stage of adipocyte development or differentiation status. By isolating preadipocytes from U-IRX3^ov^ mice and inducing brown/beige adipogenesis *in vitro*, we found that h*IRX3* overexpression following the initiation of *Ucp1* mRNA expression could effectively enhance the thermogenic capacity and *Ucp1* expression of mature brown/beige adipocytes. Nevertheless, the RNA-seq results showed that genes that were upregulated following h*IRX3* overexpression were primarily enriched in processes such as brown cell differentiation and oxidative phosphorylation, further supporting a promotive role for h*IRX3* in energy expenditure.

The overexpression of h*IRX3* either from an early stage of life or only in adulthood promoted the thermogenic potential of brown/beige adipocytes, which was consistent with our previous findings *in vitro* (16). However, other studies have indicated that IRX3 may be a negative regulator of thermogenesis through central or peripheral regulation (9, 12). Nobrega et al. previously demonstrated that obesity-associated *FTO* variants were positively associated with *IRX3* expression in the brain (12), and observed a 25%–30% decrease in body weight in *Irx3* global knockout mice fed a normocaloric diet (NCD) compared with wild-type controls, which was attributable to a significant increase in brown/beige adipocyte function. Additionally, the hypothalamic overexpression of a dominant–negative form of mouse *Irx3* (*Ins2-*Cre*;Irx3*DN) using EnR*-Irx3*, in which the EnR element was employed to inactivate *Irx3* expression, also resulted in a prominent increase in the thermogenic capacities of brown/beige adipocytes. A different group subsequently identified that rs1421085, a leading *FTO* gene variant, disrupted a conserved binding motif for the ARID5B repressor, leading to the specific disinhibition of *IRX3* expression in preadipocytes (not in mature white adipocytes) and, eventually, to the repression of preadipocyte thermogenic capacity or the impairment of adipocyte development at a very early stage of induction (day 2) *in vitro* (9). To support this, the authors generated adipose-specific *Irx3* dominant–negative (*aP2-*Cre*;Irx3*DN) mice and found phenotypes similar to those of *Ins2-*Cre*;Irx3*DN mice. Both studies supposed a negative role for *Irx3* in thermogenesis.

Interestingly, both groups crossed *Rosa26*-loxP-stop-loxP-EnR-*Irx3*DN (a theoretical dominant–negative form of Irx3) with corresponding Cre tool mice to produce the tissue-specific “knockout” models (9, 12), which could be intriguing and tricky. First, mice from both the *aP2-*Cre;*Irx3*DN and *Ins2-*Cre*;Irx3*DN lines appeared to be markedly smaller compared with controls in early life (even at three weeks), and showed a large difference (approximately 10 g) in body weight at eight weeks of age (9, 12). This indicated that these mice likely had growth impairment or developmental defects, and the increased thermogenic capacities of the evaluated adipose tissues might have been due to impaired development (32). Furthermore, the blotting of hypothalamic proteins showed concomitant similar expression levels of endogenous wild-type *Irx3* and exogenous EnR*-Irx3* in *Ins2-*Cre*;*EnR*-Irx3* mice. Indeed, the phenotypes of the *Ins2-*Cre*;*EnR*-Irx3* mice were similar to those of U-IRX3^ov^ mice, in which we employed *Rosa26*-loxP-stop-loxP-h*IRX3* using a similar strategy to that used by the Nobrega group, and where the mice displayed a reduced less body weight and increased thermogenesis in adipose tissue. However, no short stature was observed among either U-IRX3^ov^ or iU-IRX3^ov^ mice. Notably, in a different study, the authors infected isolated neonatal ventricular myocytes (NVMS) with adenovirus encoding wild-type *Irx3*, a dominant *Irx3* activator (VP16*-Irx3*), or a dominant *Irx3* repressor (EnR*-Irx3*), and found that *Cx40*/*Gja5*, an IRX3 target gene, was significantly upregulated following the overexpression of wild-type *Irx3* and EnR*-Irx3*, but not VP16*-Irx3* (13). These results raised the possibility that, under certain conditions, EnR*-Irx3* may exert a wild-type *Irx3*-like function. To address this, adipocyte-specific *Irx3* knockout models (without growth defects) using floxed *Irx3* would be beneficial for elucidating the roles of endogenous *Irx3* in thermogenesis.

Mellgren et al., who first identified a role for rs1421085 in the regulation of *IRX3* expression (9), further showed that the constitutive and complete absence of endogenous *Irx3* in embryonic fibroblasts leads to the loss of adipogenic differentiation capacity (17). ME3 cells lacking *Irx3* cannot initiate differentiation, and show profoundly inhibited mitochondrial respiration and a significant decrease in *Ucp1* and *Pgc-1α* levels when treating with brown adipocyte induction protocol (17). Accordingly, shRNA-induced *Irx3* knockdown in preadipocytes from both iWAT and BAT significantly repressed the thermogenic capacity and *Ucp1* expression in induced mature brown/beige adipocytes (at differentiation days 6–8) (16). However, the same group previously reported that silencing *Irx3* in wild-type preadipocytes derived from noncarriers (of rs1421085) did not inhibit *Ucp1* expression and thermogenic capacity at differentiation day 2, at which stage *Ucp1* expression and lipid droplet formation were almost undetectable (14). The thermogenesis phenotypes of preadipocytes on different induction day could be in different condition, which would be much essential and valuable, the detailed mechanism underlying these discrepancies was needed to be studied discreetly. Additionally, differences in species, cell lines, induction cocktail composition, *IRX3* dosage, and genetic background used by different groups are likely to lead to different outcomes.

In addition, another group presented evidence showing that partial inhibition of hypothalamic *Irx3* by lentiviral knockdown led to diet-induced adiposity, possibly through increasing caloric intake and reducing energy expenditure (15), in contrast to the metabolic phenotypes observed in *Ins2-*Cre*;*EnR*-Irx3* mice. Further research using other neuron-specific *Irx3* knockout models is required to clarify the central regulatory roles of *Irx3* in peripheral brown/beige adipocyte thermogenesis.

We previously reported that IRX3 directly binds to the *Ucp1* promoter and enhances its transcription *in vitro*. Consistent with this result, in this study, we further showed that *Ucp1* expression was increased n h*IRX3*-overexpressing brown adipocytes *in vivo*, as was the expression of several other thermogenesis-related genes, including *Pgc-1α*, *Cidea*, and *Dio2*. Whether IRX3 can regulate other genes, including the above genes that form part of the transcription complex that binds to the enhancer region of *Ucp1* to increase thermogenesis, remains to be clarified. Moreover, RNA-seq analysis revealed a marked upregulation in the expression of mitochondria-related (such as *Cox7a1*, *Cox8b*, and *Uqcrb*) and lipid metabolism-related (such as *Scd1* and *Acaa2*) genes under the condition of h*IRX3* overexpression.

In summary, our research revealed that h*IRX3* exerts a regulatory role in energy homeostasis by promoting thermogenesis in brown/beige adipose tissues. Adult humans have active depots of BATs, while the size and activity of BAT depots in obese individuals are largely reduced (33–36). Whether *FTO* variants (including rs1421085) contribute to thermogenesis, and the exact role of *IRX3* in this process, merit further and urgent investigation.

## Data Availability Statement

The data presented in the study are deposited in online repositories, accession number can be found in the article/**Supplementary Material**.

## Ethics Statement

The animal study was reviewed and approved by Animal Care Committee of Shanghai Jiao Tong University School of Medicine.

## Author Contributions

JW, JH, and RL designed the experiments and supervised the study. ZZ, QW, YH, PL, DL, and MY carried out the animal and molecular experiments. ZZ analyzed the data. ZZ and JW wrote the manuscript. WG and JH contributed to text revision and discussion. All authors contributed to the article and approved the submitted version.

## Funding 

This work was supported by grants from National Key Research and Development Program of China (2018YFC1313802), the National Natural Science Foundation of China (91957124 and 81822009), and the Outstanding Academic Leader Project of Shanghai Municipal Health Commission (2018BR01).

## Conflict of Interest

The authors declare that the research was conducted in the absence of any commercial or financial relationships that could be construed as a potential conflict of interest.

## Supplementary Material

Supplementary Figure 1 Generation of *Ucp1-*Cre^ERT2^; *Rosa26* ^h^ *^IRX3^*(iU-IRX3^ov^) mouse model. **(A)** Final Targeting Vector. Linearization site: *Not*I. **(B)** The sequence of the final targeting vector. **(C)** Overview of the targeting strategy. **(D)** DNA PCR screen of *Ucp1-*Cre^ERT2^ mice. Recombinant allele target band: 413bp; WT allele target band: 139bp. **(E)** Cre mRNA expression in varies tissue (relative to 36B4, normalized to control mice, n=4~6). **(F)** IHC for BAT UCP1 expression in *Rosa26* ^wild type(WT)^;*Ucp1-*Cre^ERT2^ (control) male mice after 7-days cold exposure. GFP antibody (CST 2956s, 1:200). Photos were taken by LSM710 (Zeiss, 40×). **(G)** Ucp1 protein expression in BAT of control and *Ucp1-*Cre^ERT2^ mice (Het in 3^rd^ lane, HO in 4^th^ lane). **(H)** The mRNA expression levels of hIRX3(top) and mIRX3 (below) in BAT, iWAT, eWAT of male iU-IRX3^ov^ and control mice after CL316,243 and TMX injection (n = 9~10).**(I-K)** The protein expression levels of hIRX3 in BAT **(I)**, iWAT **(J)** and eWAT **(K)** of male iU-IRX3^ov^ and control mice after CL316,243 and TMX injection (n = 3~5).

Supplementary Figure 2 Growth curve, body weight, physical activities and cold tolerance test in U-IRX3^ov^ mice. **(A, B)** Growth curve of NCD fed U-IRX3^ov^ and control from 4 weeks to 10 weeks. **(A)** Male body weight (n=6~8). **(B)** Female body weigth (n=9~11). **(C-F)** Body weight **(C)**, fat mass percentage **(D)**, lean mass percentage **(E)** and average food intake **(F)** of female U-IRX3^ov^ and Ctrl (n = 9~11, average of 3 individual measurements). **(G-I)** 24-hour (left) and total (right) physical activities on X-axis **(G)**, Y-axis **(H)** and Z-axis **(I)** of male U-IRX3^ov^ and control mice. **(J)** Body temperature changes of male U-IRX3^ov^ and control mice (n=9~10) for the first 6 h in 4°C cold room. **(K)** The mRNA expression levels of hIRX3 (left) and mIRX3 (right) in BAT, iWAT and eWAT of male U-IRX3^ov^ and control mice after cold stimulation (n = 9~10).

Supplementary Figure 3 Overexpression of hIRX3 from the embryonic stage enhances β3-AR agonist-induced thermogenesis in female U-IRX3^ov^ mice **(A-H).** Tissue mass percentage **(A)**, representative images of HE staining of BAT (top), iWAT (middle) and gWAT (bottom) **(B)** and adipocyte size distribution of iWAT**(C)**, gWAT **(D)** of 10-week-old female U-IRX3^ov^ and control mice after 1-h CL 316,243 (1.5 mg/kg body weight) injection, the mRNA expression levels of thermogenesis-related genes (n = 9~11) **(E)**, the protein levels of Ucp1 and Pgc-1α in iWAT (n = 3) **(F)**, the mRNA expression levels of thermogenesis-related genes (n = 6~8) **(G)**, and the protein levels of Ucp1 and Pgc-1α in gWAT (n = 3) **(H)**. Expression level of hIRX3 mRNA **(I)** and mIRX3 mRNA **(J)** in SVF of male U-IRX3^ov^ mice BAT(n=6) and iWAT n=(4). Scale bars, 100 μm. Data are shown as mean ± SEM. *P < 0.05, **P < 0.01, ***P < 0.001.

Supplementary Table 1 Mouse and human qPCR primers sequences.

Supplementary Table 2 All down regulated genes in RNA-sequencing analysis in **Figure 5**.

Supplementary Table 3 All up regulated genes in RNA-sequencing analysis **Figure 5**.
