# Supplementary figures and images for: IRX3 Overexpression Enhances Ucp1 Expression In Vivo
Source: Front Endocrinol (Lausanne). 2021 Mar 10;12:634191. doi: 10.3389/fendo.2021.634191 (PMC7988233; doi:10.3389/fendo.2021.634191)

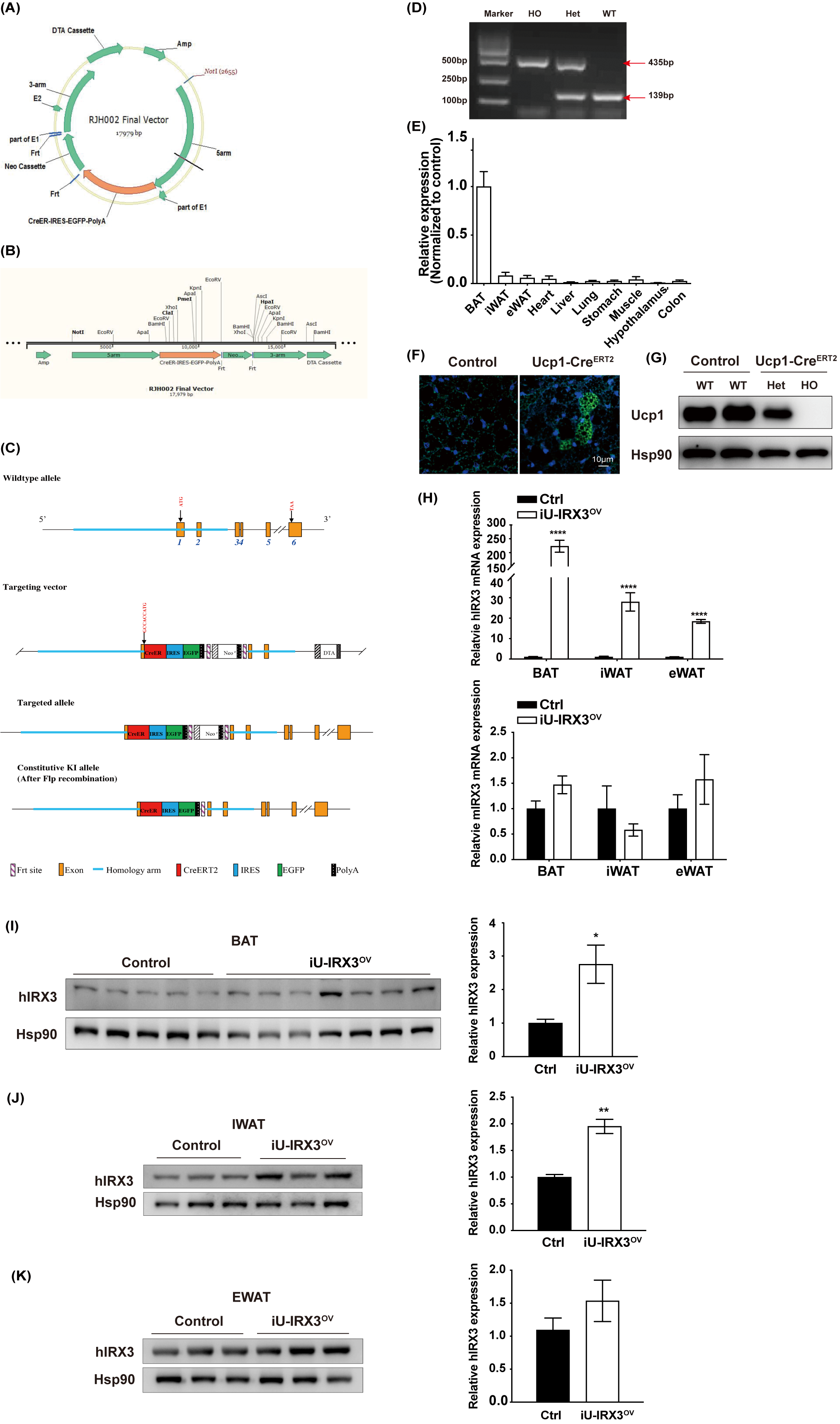

Supplement: Supplementary Figure 1 — Generation of Ucp1-CreERT2; Rosa26 h IRX3(iU-IRX3ov) mouse model. (A) Final Targeting Vector. Linearization site: NotI. (B) The sequence of the final targeting vector. (C) Overview of the targeting strategy. (D) DNA PCR screen of Ucp1-CreERT2 mice. Recombinant allele target band: 413bp; WT allele target band: 139bp. (E) Cre mRNA expression in varies tissue (relative to 36B4, normalized to control mice, n=4~6). (F) IHC for BAT UCP1 expression in Rosa26 wild type(WT);Ucp1-CreERT2 (control) male mice after 7-days cold exposure. GFP antibody (CST 2956s, 1:200). Photos were taken by LSM710 (Zeiss, 40×). (G) Ucp1 protein expression in BAT of control and Ucp1-CreERT2 mice (Het in 3rd lane, HO in 4th lane). (H) The mRNA expression levels of hIRX3(top) and mIRX3 (below) in BAT, iWAT, eWAT of male iU-IRX3ov and control mice after CL316,243 and TMX injection (n = 9~10).(I-K) The protein expression levels of hIRX3 in BAT (I), iWAT (J) and eWAT (K) of male iU-IRX3ov and control mice after CL316,243 and TMX injection (n = 3~5). [file Image_1.tif]

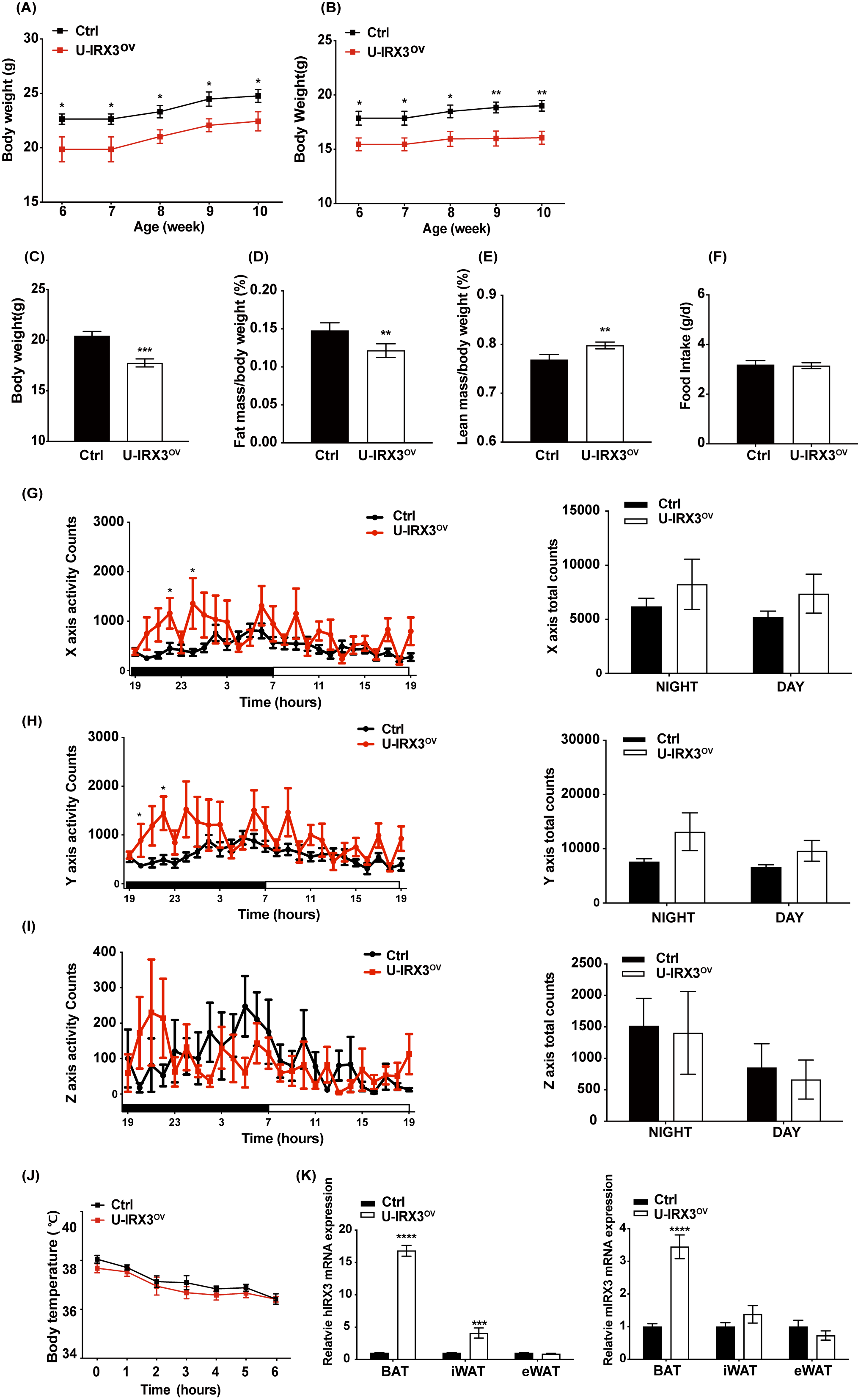

Supplement: Supplementary Figure 2 — Growth curve, body weight, physical activities and cold tolerance test in U-IRX3ov mice. (A, B) Growth curve of NCD fed U-IRX3ov and control from 4 weeks to 10 weeks. (A) Male body weight (n=6~8). (B) Female body weigth (n=9~11). (C-F) Body weight (C), fat mass percentage (D), lean mass percentage (E) and average food intake (F) of female U-IRX3ov and Ctrl (n = 9~11, average of 3 individual measurements). (G-I) 24-hour (left) and total (right) physical activities on X-axis (G), Y-axis (H) and Z-axis (I) of male U-IRX3ov and control mice. (J) Body temperature changes of male U-IRX3ov and control mice (n=9~10) for the first 6 h in 4°C cold room. (K) The mRNA expression levels of hIRX3 (left) and mIRX3 (right) in BAT, iWAT and eWAT of male U-IRX3ov and control mice after cold stimulation (n = 9~10). [file Image_2.tif]

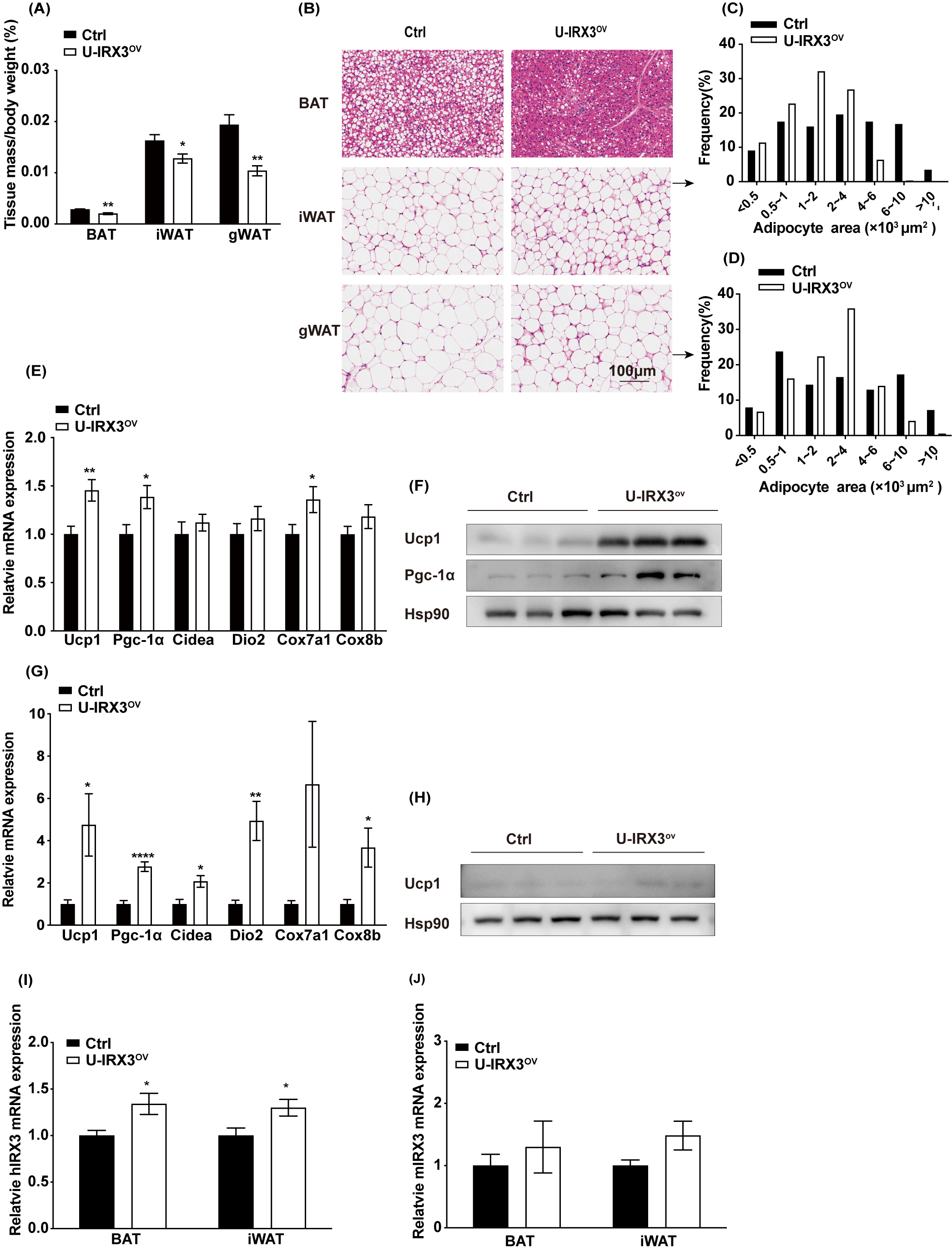

Supplement: Supplementary Figure 3 — Overexpression of hIRX3 from the embryonic stage enhances β3-AR agonist-induced thermogenesis in female U-IRX3ov mice (A-H). Tissue mass percentage (A), representative images of HE staining of BAT (top), iWAT (middle) and gWAT (bottom) (B) and adipocyte size distribution of iWAT(C), gWAT (D) of 10-week-old female U-IRX3ov and control mice after 1-h CL 316,243 (1.5 mg/kg body weight) injection, the mRNA expression levels of thermogenesis-related genes (n = 9~11) (E), the protein levels of Ucp1 and Pgc-1α in iWAT (n = 3) (F), the mRNA expression levels of thermogenesis-related genes (n = 6~8) (G), and the protein levels of Ucp1 and Pgc-1α in gWAT (n = 3) (H). Expression level of hIRX3 mRNA (I) and mIRX3 mRNA (J) in SVF of male U-IRX3ov mice BAT(n=6) and iWAT n=(4). Scale bars, 100 μm. Data are shown as mean ± SEM. *P < 0.05, **P < 0.01, ***P < 0.001. [file Image_3.tif]
